# The influence of age, sex, and mandibular morphometric parameters on cortical bone width and erosion: a panoramic radiography study

**DOI:** 10.3389/fdmed.2025.1558372

**Published:** 2025-03-26

**Authors:** Bramma Kiswanjaya, Shafira Ramadhanti Taufiq, Syurri Innaddinna Syahraini, Akihiro Yoshihara

**Affiliations:** ^1^ Department of Dentomaxillofacial Radiology, Faculty of Dentistry, University of Indonesia, Jakarta, Indonesia; ^2^ Division of Oral Science for Health Promotion, Department of Oral Health Science and Promotion, Graduate School of Medical and Dental Sciences, Niigata University, Niigata City, Japan

**Keywords:** mandibular cortical width, mandibular cortical index, panoramic radiography, mandibular morphology, age, sex

## Abstract

**Aim:**

This study evaluated the relationship between age, sex, and mandibular morphological changes, focusing particularly on mandibular cortical width (MCW) and mandibular cortical index (MCI), using panoramic radiography.

**Methods:**

A total of 300 panoramic radiographs were analyzed. Mandibular morphometric parameters were measured, including ramus height, maximum and minimum ramus width, gonial angle, bigonial width, condylar height, coronoid height, MCW, and MCI. Statistical analysis included Spearman's correlation, multiple regression, and logistic regression to assess the relationships between mandibular morphology, cortical width, and cortical erosion with age and sex.

**Results:**

Significant differences were observed across age and sex groups in most mandibular parameters. Men had significantly larger values for ramus height, bigonial width, and condylar height, while women showed a wider gonial angle. Age was negatively correlated with MCW (*r* = −0.522, *p* = 0.000) and positively correlated with MCI (*r* = 0.388, *p* = 0.000), indicating that cortical width decreases and cortical erosion increases with age. In the multiple regression analysis, age (*B* = −0.028, *p* = 0.000) and MCI (*B* = −0.391, *p* = 0.000) were strong negative predictors of MCW. In contrast, condylar height (*B* = 0.024, *p* = 0.007) positively influenced MCW, explaining 41.5% of the variance in cortical width (*R*² = 0.415). The logistic regression analysis revealed that MCW [Exp(*B*) = 0.157, *p* = 0.000], sex [Exp(*B*) = 2.251, *p* = 0.005], and age [Exp(*B*) = 1.062, *p* = 0.000] significantly predicted MCI, with thinner mandibular cortices, female sex, and older age associated with higher MCI values (severe cortical erosion). Each 1 mm decrease in MCW increased the likelihood of being in a higher MCI class by 84%.

**Conclusions:**

Women were more than twice as likely to exhibit greater cortical erosion than men. This study demonstrated that age, sex, and mandibular morphometric parameters significantly influenced MCW and erosion.

## 1 Introduction

Panoramic radiographs (orthopantomograms) are commonly used for diagnostic purposes (1). These radiographs provide valuable information about mandibular morphology and can potentially be utilized for osteoporosis screening (2). Two indices, the MCW and the mandibular cortical index (MCI), have been proposed as radiographic markers for evaluating cortical bone changes related to osteoporosis (3, 4). MCW measures the width of the mandibular cortex below the mental foramen, while MCI categorizes cortical erosion into three classes based on the appearance of the mandibular cortex. These indices have shown potential in identifying individuals at risk of osteoporosis (5), particularly when considered alongside mandibular morphometric parameters, such as ramus height, condylar height, and gonial angle (6).

Research has demonstrated significant variations in mandibular morphology associated with age and sex, which are also important factors in osteoporosis progression (7). Older individuals and women, particularly postmenopausal women, are more likely to experience cortical erosion and changes in mandibular dimensions (8). Several studies have shown that reductions in mandibular cortical width (MCW) and changes in gonial angle can be correlated with systemic bone loss (9). However, the exact relationship between mandibular morphometric parameters, cortical erosion (MCI), and cortical width (MCW) has yet to be fully explored comprehensively.

This study evaluated the relationship between age, sex, and mandibular morphological changes, including MCW and MCI, in a population undergoing panoramic radiography. Various mandibular dimensions were analyzed, such as ramus height, gonial angle, condylar height, and bigonial width. This research aimed to determine whether these parameters, along with age and sex, can serve as reliable indicators of cortical bone quality and potential osteoporosis risk. Through multiple regression and correlation analyses, we investigated the role of mandibular morphometry in osteoporosis detection, contributing to the ongoing effort to develop cost-effective screening tools for early osteoporosis diagnosis.

## 2 Materials and methods

### 2.1 Study design and sample selection

This cross-sectional study utilized panoramic radiographs of patients aged 45–85 years. A total of 300 radiographs were selected, comprising an equal number of men (*n* = 150) and women (*n* = 150). The radiographs were retrospectively collected starting in October 2023, and consecutive sampling was used until the required sample size was reached. Digital panoramic radiographs were obtained using the Veraviewepocs 2D system and i-Dixel imaging software (J. Morita Corp., Kyoto, Japan). Imaging parameters included an exposure setting of 10 milliampere-seconds (mAs) for 12–15 s at 70–80 kVp. Radiographs were selected based on specific inclusion and exclusion criteria.

The inclusion criteria included the following: radiographs met high-quality standards, with minimal vertical, horizontal, and anteroposterior angulation errors; a clearly visible region of interest (ROI) in the measured areas; and patients within the age range of 45–85 years with no history of osteoporosis treatment or significant craniofacial trauma. The exclusion criteria included the following: radiographs with evidence of fractures or pathological conditions affecting the measured areas; patients with a history of systemic diseases that could impact bone metabolism, such as diabetes, thyroid disorders, or long-term steroid use; and radiographs of patients with congenital abnormalities or syndromes affecting the jaw structure. Ethical approval for the study was obtained from the Faculty of Dentistry, Universitas Indonesia (protocol No. 010670823), with letter No. 44/Ethical Approval/FKGUI/IX/2023. The study was conducted in accordance with the Declaration of Helsinki. A clear written consent informing about the procedure and the use of radiographs for scientific use, was obtained from patients to ensure their knowledge about participating in this study.

### 2.2 Parameters measured

**Figure 1:**
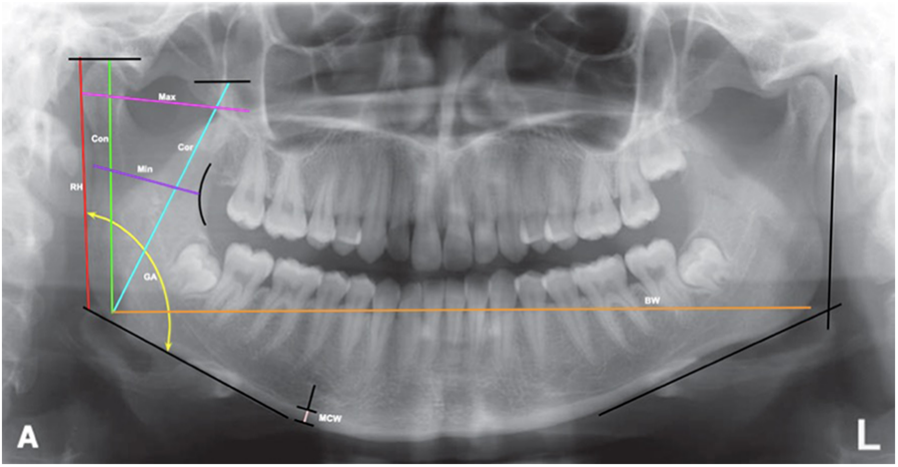
Measurement of mandibular parameters [ramus height (RH), maximum ramus width (max), minimum ramus width (min), gonial angle (GA), bigonial width (BW), condylar process height (Con), coronoid process height (Cor), and mandibular cortical width (MCW)] on panoramic radiographs.

**Figure 2:**
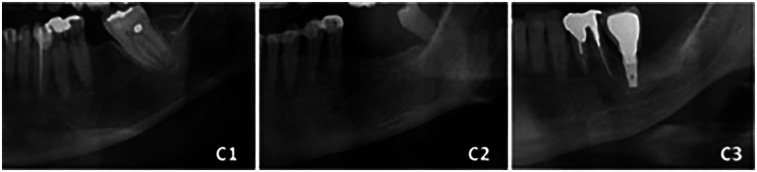
Mandibular cortical index (MCI) categories: (C1) class 1 (normal cortex); (C2) class 2 (mild to moderate cortical erosion); (C3) class 3 (severe cortical erosion).

This study focused on nine mandibular morphometric parameters, which were measured using panoramic radiographs: parameters 1–8 are shown in Figure 1, while parameter 9 (MCI) is illustrated in Figure 2.

1. Ramus height (RH): The distance from the most superior lateral point to the most inferior lateral point of the ramus.
2. Maximum ramus width (Max): The distance between the most anterior and posterior points of the ramus passing through the sigmoid notch.
3. Minimum ramus width (Min): The shortest anterior–posterior distance of the ramus.
4. Gonial angle (GA): The angle formed between the ramus and mandibular body, determined by the intersection of the inferior border of the mandible and the posterior border of the ramus and condylar process.
5. Bigonial width (BW): The distance between two gonion points (Go), the most inferior, posterior, and lateral points at the external angle of the mandible.
6. Condylar height (Con): The distance from the highest point of the condylar process to the intersection of the orientation line and the inferior border of the ramus.
7. Coronoid height (Cor): The distance from the highest point of the coronoid process to the intersection of the orientation line and the inferior border of the ramus.
8. Mandibular cortical width (MCW): The distance between the inferior and superior cortices of the mandible below the mental foramen region.
9. Mandibular cortical index (MCI): The cortical shape of the mandible on panoramic radiographs, located distal to the mental foramen, is categorized into three classes according to the Klemetti index:
  • Class 1 (normal cortex): Smooth and sharp endosteal margins of the cortex on both sides
  • Class 2 (mild to moderate erosion): The endosteal margin shows semilunar defects (lacunar resorption) or moderate endosteal cortical remnants
  • Class 3 (severe erosion): The cortical layer exhibits severe endosteal cortical remnants and appears porous

Parameters 1–8 were measured on the side of the mandible that was most clearly visible, as previous research has shown no statistically significant differences between the right and left sides of the mandible (10, 11). For parameter 9, the MCI, if there was a discrepancy between the right and left sides, the side with the more severe MCI classification was selected.

### 2.3 Reliability of measurements

To ensure accuracy and consistency, intra- and inter-observer reliability was evaluated using intraclass correlation coefficients (ICC) for the numerical data ramus height (RH) and Kappa agreement for the categorical data (MCI) on a random sample of 50 panoramic radiographs. The first set of measurements was performed by a 4th-year dental student who had undergone two weeks of training and calibration, with a minimum of 30 min per session, focusing on mandibular morphology measurement, MCW assessment, and MCI classification. The second set of measurements was conducted by a dentomaxillofacial radiology lecturer with 15 years of experience interpreting radiomorphometric indices.

### 2.4 Statistical analysis

All statistical analyses were performed using the Statistical Program for the Social Sciences (SPSS) version 22 for Windows (IBM Corporation). The significance level was set at *p* < 0.05. Descriptive statistics were calculated for all measured parameters, including the means, standard deviations, and ranges. Differences across age and sex groups were assessed using the independent samples *T*-test (for normally distributed continuous variables) or the Mann–Whitney *U* test (for non-normally distributed variables). Spearman's correlation was used to assess the relationships between age, sex, and mandibular morphometric parameters with MCW and MCI. The correlation coefficients and corresponding *p*-values were reported. A multiple regression analysis (stepwise method) was conducted with MCW as the dependent variable and age, MCI, and condylar height as independent variables. The results were presented with unstandardized coefficients (*B*), standardized coefficients (Beta), *t*-values, and *R*-squared values to assess how much variance in MCW was explained by these factors. A logistic regression analysis (forward stepwise method) was performed using MCI (categorized as Class 1 vs. Class 2 & 3) as the dependent variable and MCW, age, and sex as independent variables. Odds ratios [Exp(*B*)], confidence intervals, and *p*-values were reported to determine the likelihood of higher MCI classes.

## 3 Results

The results of intra-and inter-observer showed high reliability, with an ICC of 0.95 for intra-observer agreement and 0.92 for inter-observer agreement for RH, indicating excellent consistency in the numerical measurements. For the categorical data (MCI), the Kappa agreement was 0.87 for intra-observer reliability and 0.78 for inter-observer reliability, suggesting substantial agreement in the classification of MCI. These values reflect the robustness of the measurement protocol and suggest that the training and experience levels of the observers contributed to the high reliability of the measurements. The near-perfect ICC and strong Kappa values indicated minimal variability between observers, ensuring confidence in the reproducibility and accuracy of the results obtained in this study.

Table 1 summarizes the descriptive statistics of the mandibular morphometric parameters based on sex and age groups. Significant differences were observed across nearly all parameters. Men consistently showed larger measurements for RH, maximum and minimum ramus width, BW, condylar height, and coronoid height compared to women (*p* < 0.05). Additionally, individuals aged 45–64 years had significantly larger values for these parameters compared to those over 64 years old (*p* < 0.05). The GA was notably larger in women and in the older age group. Although there was no significant difference in MCW between sexes, younger individuals had a significantly greater MCW than older individuals (*p* < 0.001). Moreover, the MCI indicated that Class 1 (normal cortex) was more prevalent in men and younger individuals, whereas Class 3 (severe erosion) was more common in women and older adults (*p* < 0.05).

**Table 1:** Descriptive statistics of mandibular morphometric parameters by sex and age group.

Variables	Sex		*P* value	Age		*P* value
	Men (*N* = 150)	Women (*N* = 150)		45–64 years (*N* = 199)	>64 years (*N* = 101)	
	Mean ± SD	Mean ± SD		Mean ± SD	Mean ± SD	
	Median (min–max)	Median (min–max)		Median (min–max)	Median (min–max)	
Ramus height (mm)	53.2 ± 3	48.8 ± 1.6	0.000*	51.4 ± 3	50.2 ± 3.6	0.001*
	52.2 (46–61.7)	48.7 (41.8–55.5)		50.8 (47.7–61.8)	47.9 (41.8–60.9)	
Maximum ramus width (mm)	37.5 ± 1.5	35.1 ± 1.3	0.000*	36.5 ± 1.7	36 ± 2	0.008*
	37.2 (32.6–43.2)	34.9 (31–42.2)		36.2 (32.6–43.2)	34.8 (31–41.6)	
Minimum ramus width (mm)	24.9 ± 1.2	23.2 ± 1.2	0.000*	24.3 ± 1.4	23.7 ± 1.5	0.000*
	24.9 (21.1–30.2)	23 (20.4–31.4)		24 (21.1–31.4)	22.9 (20.4–29.7)	
Gonial angle (°)	119.9 ± 2.8	124.9 ± 2.8	0.000*	121.9 ± 3.5	123.4 ± 4	0.005*
	119.9 (107.1–134.8)	124.9 (108.8–134.9)		121.2 (107.1–134.8)	125.9 (112.4–134.9)	
Bigonial width (mm)	177.2 ± 4.7	169.4 ± 4.5	0.000*	174 ± 5.3	172 ± 7.1	0.002*
	176.5 (161.5–196.9)	168.5 (151.1–193)		173.7 (157.3–196.9)	167.5 (151.1–192.4)	
Condylar height (mm)	53 ± 2.9	48.4 ± 1.6	0.000*	51.1 ± 3	49.9 ± 3.6	0.002*
	52.1 (45.8–61.4)	48 (41.4–54.4)		50.4 (47.2–61.4)	47.6 (41.4–60.3)	
Coronoid height (mm)	53.6 ± 2.8	49.4 ± 1.7	0.000*	51.9 ± 2.9	50.7 ± 3.4	0.003*
	53 (49.6–65.4)	49.1 (40.7–57.4)		51.6 (45.2–65.4)	48.9 (40.7–63)	
Mandibular cortical width (mm)	3.1 ± 0.6	3 ± 0.7	NS	3.3 ± 0.5	2.6 ± 0.6	0.000**
	3 (1.6–4.7)	3 (1–4.7)		3.3 (1.6–4.7)	2.6 (1–3.8)	
Mandibular cortical index	N (%)	N (%)		N (%)	N (%)	
Class 1	83 (27.7)	58 (19.3)	0.001^	121 (40.3)	20 (6.7)	0.000^
Class 2	55 (18.3)	59 (19.7)		67 (22.3)	47 (15.7)	
Class 3	12 (4)	33 (11)		11 (3.7)	34 (11.3)	

NS, not significant.

* Independent samples Mann–Whitney *U* test.

** Independent samples *t*-test.

^ Pearson's chi-square test.

Table 2 compares mandibular morphometric parameters across different MCI classes. Significant differences were observed in all parameters between Class 1 (normal cortex), Class 2 (mild to moderate erosion), and Class 3 (severe erosion). Individuals in Class 1 exhibited the largest measurements for RH, maximum and minimum ramus width, BW, condylar height, coronoid height, and MCW (*p* < 0.05). Conversely, those in Class 3 (severe cortical erosion) consistently showed the smallest measurements across these parameters. The GA progressively increased from Class 1 (121.6 ± 3°) to Class 3 (124.4 ± 4.2°, *p* = 0.000), indicating a widening of the angle with increasing cortical erosion. MCW was also significantly reduced in Class 3 (2.2 ± 0.5 mm) compared to Class 1 (3.4 ± 0.5 mm, *p* = 0.000), reflecting the severity of bone loss. These findings suggest a clear association between mandibular cortical erosion and reductions in mandibular dimensions, with more severe erosion correlating with smaller morphometric values.

**Table 2:** Comparison of mandibular morphometric parameters across different mandibular cortical index (MCI) classes.

Variables	Mandibular cortical index			*P* value
	Class 1 (*N* = 141)	Class 2 (*N* = 114)	Class 3 (*N* = 45)	
	Mean ± SD	Mean ± SD	Mean ± SD	
	Median (min–max)	Median (min–max)	Median (min–max)	
RH (mm)	51.4 ± 3.2	51 ± 3.2	49.4 ± 3.1	0.000*
	50.8 (46–61.7)	50.7 (43.5­–61.8)	47.8 (41.8–60.8)	
Maximum ramus width (mm)	36.5 ± 1.8	36.4 ± 1.9	35.6 ± 1.7	0.000*
	36.2 (32.6–43.2)	36.23 (31–42)	34.8 (33.4–39.9)	
Minimum ramus width (mm)	24.2 ± 1.3	24.1 ± 1.5	23.2 ± 1.3	0.000*
	24.1 (22–31.4)	23.9 (20.8–30.2)	22.8 (20.4–25.8)	
Gonial angle (°)	121.6 ± 3	122.5 ± 4.2	124.4 ± 4.2	0.000*
	121.1 (111.9–129.6)	123.3 (107.1–134.8)	126 (112.4–134.9)	
Bigonial width (mm)	174.4 ± 5	173 ± 6.5	170.5 ± 7	0.000*
	174.5 (166.8–195.7)	172 (157.3–196.9)	167.3 (151.1–190.2)	
Condylar height (mm)	51.1 ± 3.2	50.8 ± 3.2	49.1 ± 3.2	0.000*
	50.7 (45.8–61.3)	50.2 (43.5–61.4)	47.6 (41.4–60.4)	
Coronoid height (mm)	52 ± 3.1	51.4 ± 3	49.9 ± 3.1	0.000*
	51.7 (47.8–65.4)	51.2 (45.2–63)	48.5 (40.7–61)	
Mandibular cortical width (mm)	3.4 ± 0.5	2.9 ± 0.5	2.2 ± 0.5	0.000**
	3.3 (2–4.7)	3 (1.9–4.5)	2.2 (1–3.2)	

* Kruskal–Wallis Test.

** One-way analysis of variance (ANOVA).

Table 3 presents Spearman's rho correlation analysis between MCI, MCW, and various factors such as age, sex, and mandibular morphometric parameters. A significant positive correlation was observed between MCI and age (*r* = 0.388, *p* = 0.000), indicating that cortical erosion increased with age. At the same time, MCW showed a significant negative correlation with age (*r* = −0.522, *p* = 0.000), reflecting decreased cortical width in older individuals. Additionally, MCI was positively correlated with sex (*r* = 0.167, *p* = 0.004), suggesting that women tended to have greater cortical erosion, although there was no significant correlation between sex and MCW. For mandibular dimensions, MCI was negatively correlated with RH (*r* = −0.116, *p* = 0.044), minimum ramus width (*r* = −0.126, *p* = 0.029), BW (*r* = −0.192, *p* = 0.001), condylar height (*r* = −0.125, *p* = 0.031), and coronoid height (*r* = −0.151, *p* = 0.009), indicating that greater cortical erosion (higher MCI values) is associated with smaller mandibular dimensions. Conversely, MCW was positively correlated with RH (*r* = 0.125, *p* = 0.03), BW (*r* = 0.172, *p* = 0.003), condylar height (*r* = 0.134, *p* = 0.02), and coronoid height (*r* = 0.13, *p* = 0.024), showing that thicker mandibular cortices are associated with larger morphometric measurements. Both MCI and MCW were significantly correlated with GA (*p* < 0.05), with MCI showing a positive and MCW showing a negative correlation, indicating that increased GA is related to greater cortical erosion and thinner cortices.

**Table 3:** Correlation of mandibular cortical Index and mandibular cortical width with age, sex, and morphometric parameters using spearman's rho test.

Variables	Mandibular cortical index (1: class 1; 2: class 2 & 3)		Mandibular cortical width (mm)	
	Correlation coefficient	*P* value	Correlation coefficient	*P* value
Age (1:45–64 years; 2: >64 years)	0.388	0.000	−0.522	0.000
Sex (1: men; 2: women)	0.167	0.004	−0.067	NS
Ramus height (mm)	−0.116	0.044	0.125	0.03
Maximum ramus width (mm)	−0.08	NS	0.081	NS
Minimum ramus width (mm)	−0.126	0.029	0.101	NS
Gonial angle (°)	0.183	0.001	−0.115	0.047
Bigonial width (mm)	−0.192	0.001	0.172	0.003
Condylar height (mm)	−0.125	0.031	0.134	0.02
Coronoid height (mm)	−0.151	0.009	0.13	0.024
Mandibular cortical width (mm)	−0.529	0.000	—	—
Mandibular cortical index (1: class 1; 2: class 2 & 3)	—	—	−0.529	0.000

Table 4 presents the results of a stepwise multiple regression analysis using MCW as the dependent variable, where age, MCI, and condylar height were identified as significant predictors. Age had a strong negative association with MCW (*B* = −0.028, *p* = 0.000), indicating decreased cortical width as individuals age. Likewise, the MCI (comparing Class 1 to Classes 2 & 3) also showed a significant negative relationship with MCW (*B* = −0.391, *p* = 0.000), suggesting that individuals with greater cortical erosion (higher MCI classes) exhibit thinner mandibular cortices. Conversely, condylar height was positively associated with MCW (*B* = 0.024, *p* = 0.007), implying that individuals with a higher condylar height tend to have thicker mandibular cortices. The final regression model explained 41.5% of the variance in MCW (*R*^2^ = 0.415), with age being the most influential predictor (*β* = −0.423), followed by MCI (*β* = −0.306) and condylar height (*β* = 0.123).

**Table 4:** Multiple regression analysis (stepwise method) of factors influencing mandibular cortical width.

Mandibular cortical width (mm)	Unstandardized coefficients		Standardized coefficients	*T*	*P* value	*R*	R square
Model	*B*	Standard error	*β*				
1. (Constant)	5.262	0.192		27.346	0.000	0.562^a^	0.315
Age (years old)	−0.037	0.003	−0.562	−11.717	0.000		
2. (Constant)	5.332	0.181		29.492	0.000	0.632^b^	0.400
Age (years old)	−0.028	0.003	−0.420	−8.395	0.000		
Mandibular cortical index (1: class 1; 2: class 2 & 3)	−0.413	0.064	−0.323	−6.463	0.000		
3. (Constant)	4.093	0.486		8.415	0.000	0.644^c^	0.415
Age (years old)	−0.028	0.003	−0.423	−8.554	0.000		
Mandibular cortical index (1: class 1; 2: class 2 &3)	−0.391	0.064	−0.306	−6.136	0.000		
Condylar height (mm)	0.024	0.009	0.123	2.739	0.007		

^a^ Predictor: (constant), age (years old).

^b^ Predictor: (constant), age (years old), mandibular cortical index (1: class 1; 2: class 2 & 3).

^c^ Predictor: (constant), age (years old), mandibular cortical index (1: class 1; 2: class 2 & 3), condylar height (mm).

Table 5 presents the results of a forward stepwise logistic regression analysis using MCI as the dependent variable, where MCW, sex, and age were identified as significant predictors of MCI. MCW showed a strong negative association with MCI [*B* = −1.852, Exp(*B*) = 0.157, *p* = 0.000], indicating that individuals with thinner mandibular cortices are significantly more likely to fall into higher MCI classes, which reflects greater cortical erosion. Sex was also a significant predictor [*B* = 0.811, Exp(*B*) = 2.251, *p* = 0.005], with women being more than twice as likely as men to exhibit greater cortical erosion (higher MCI classes). Furthermore, age was positively associated with MCI [*B* = 0.061, Exp(*B*) = 1.062, *p* = 0.000], indicating that older individuals are more likely to have higher MCI values. The odds ratios [Exp(*B*)] reveal that for each 1 mm decrease in MCW, the likelihood of being in a higher MCI class increases by approximately 84% [Exp(*B*) = 0.157], while older age and female sex also substantially increase the odds of cortical erosion.

**Table 5:** Logistic regression analysis [forward stepwise (conditional) method] of factors predicting the mandibular cortical Index.

Mandibular cortical index (1: class 1; 2: class 2 & 3)	*B*	S.E.	Wald	Exp (*B*)	*P* value
Step 1^a^					
Mandibular cortical width (mm)	−2.3	0.305	56.824	0.1	0.000
Constant	7.182	0.955	56.534	1,316.078	0.000
Step 2^b^					
Mandibular cortical width (mm)	−1.833	0.328	31.311	0.16	0.000
Age (years old)	0.057	0.017	11.006	1.058	0.001
Constant	2.355	1.685	1.953	10.536	0.162
Step 3^c^					
Mandibular cortical width (mm)	−1.852	0.336	30.367	0.157	0.000
Sex (1: men; 2: women)	0.811	0.286	8.027	2.251	0.005
Age (years old)	0.061	0.017	12.126	1.062	0.000
Constant	0.989	1.772	0.312	2.690	0.577

^a^ Variable(s) entered on step 1: Mandibular cortical width (mm).

^b^ Variable(s) entered on step 2: Age (years old).

^c^ Variable(s) entered on step 3: Sex (1: men; 2: women).

## 4 Discussion

The findings of this study highlight the significant influence of age, sex, and mandibular morphometric parameters on MCW and MCI. These results are consistent with previous studies demonstrating that mandibular morphology and cortical bone changes are associated with systemic bone loss, particularly in older adults and women (12). Our results emphasize the utility of panoramic radiography as a tool for identifying individuals at risk for osteoporosis by assessing mandibular cortical changes (1–5).

The significant negative correlation between age and MCW (*r* = −0.522, *p* = 0.000) shown in Table 3 suggests that cortical thinning in the mandible is more pronounced in older individuals. This is consistent with findings of other studies indicating that bone mineral density (BMD) decreases with age, particularly in postmenopausal women, due to reduced estrogen levels, which play a crucial role in maintaining bone density (13). In our regression analysis (Table 4), age was a strong predictor of MCW, reinforcing our finding that mandibular cortical width diminishes with advancing age. Moreover, the significant positive correlation between age and MCI (*r* = 0.388, *p* = 0.000) supports the view that cortical erosion increases with age, further reflecting systemic bone loss (8). Our data also showed significant differences in various mandibular morphometric parameters across age groups (Table 1), with older individuals exhibiting smaller RH, BW, condylar height, and a wider GA. These changes in mandibular morphology may result from reduced bone mass, leading to resorptive changes in the mandibular structure (14). Such findings are supported by previous research indicating that bone loss in the mandible mirrors skeletal osteoporosis, and panoramic radiography may provide valuable insight into systemic bone health (1–4).

Sex differences in MCW and erosion were evident in our analysis. Women had significantly higher MCI values, indicating more cortical erosion compared to men, consistent with the well-established higher prevalence of osteoporosis among women, particularly postmenopausal women (8, 11). The logistic regression analysis (Table 5) demonstrated that women were twice as likely to have higher MCI values [Exp(*B*) = 2.020, *p* = 0.012], which is consistent with previous research highlighting that women are at greater risk for cortical bone degradation due to hormonal changes (8, 11). Furthermore, sex differences were observed in various mandibular morphometric parameters. Men consistently exhibited greater measurements for RH, BW, and condylar height than women (Table 1). This is consistent with the findings of other studies, which suggest that men generally have higher bone masses and larger mandibular structures due to greater overall skeletal size and bone density (15). However, women tend to have a wider GA, which may be related to the resorption of mandibular bone due to osteoporosis, particularly in older women (16).

Our study further revealed significant correlations between MCI and various mandibular morphometric parameters, with higher MCI values (indicating greater cortical erosion) associated with smaller RH, condylar height, BW, and a larger GA (Table 3). In contrast, Table 2 shows that as MCI increases from Class 1 to Class 3, there is a significant reduction in RH, maximum ramus width, minimum ramus width, condylar height, and coronoid height, along with a progressive increase in GA, suggesting that greater cortical erosion leads to reduced mandibular dimensions and reflects the systemic effects of osteoporosis on mandibular bone structure. These trends reinforce the notion that greater cortical erosion is associated with smaller mandibular dimensions, which may reflect the systemic effects of osteoporosis on mandibular bone structure. This relationship further emphasizes the value of MCI as a marker of systemic bone health (17).

In contrast, individuals with higher MCW values, indicative of wider mandibular cortices, exhibited a greater RH, BW, and condylar height (Table 3). This suggests that a wider cortical bone is associated with more robust mandibular structures, supporting the idea that mandibular morphometry can indicate overall bone health. Additionally, the negative correlation between MCW and GA (*r* = −0.115, *p* = 0.047) suggests that individuals with thinner cortices tend to have a wider GA, which may be related to resorptive changes in the mandible due to osteoporosis (18).

The strong associations between mandibular cortical changes, age, sex, and mandibular morphometric parameters observed in this study suggest that panoramic radiography could be an effective tool for early osteoporosis screening. Dual-energy x-ray absorptiometry (DXA) is recommended only for women over 65, men over 70, and individuals over 50 with fractures from minimal trauma (19). Furthermore, DXA is not always accessible, especially in dental clinics. Panoramic radiographs offer a practical alternative for identifying at-risk patients based on MCW and MCI patterns. Prior research supports the use of MCW and MCI as markers of systemic bone loss, and our findings further validate their utility (20, 21). Identifying at-risk individuals through panoramic radiographs can prompt further evaluation using DXA, potentially reducing the incidence of osteoporotic fractures.

The findings of this study highlight the potential clinical application of mandibular cortical width (MCW) and mandibular cortical index (MCI) as screening tools for osteoporosis in dental settings. Since osteoporosis is often asymptomatic until fractures occur, early detection is crucial for preventing disease progression and complications. Our results indicate that older individuals and women exhibit greater cortical erosion and reduced cortical width, consistent with systemic osteoporosis-related bone loss. Given that panoramic radiography is widely available in dental clinics, incorporating MCW and MCI assessments into routine dental examinations could serve as an adjunctive screening method to identify individuals at risk of osteoporosis. Furthermore, integrating this approach into clinical practice may facilitate timely referrals for further evaluation using dual-energy x-ray absorptiometry (DXA), allowing early diagnosis and intervention. Patients identified with high-risk mandibular cortical changes could benefit from lifestyle modifications, dietary supplementation, or pharmacological interventions to mitigate bone loss. The implementation of such a screening strategy in dentistry has the potential to bridge the gap between oral health and systemic disease management, contributing to a multidisciplinary approach for osteoporosis prevention and treatment.

While our study provides valuable insights into the relationships between mandibular morphology, cortical bone changes, and systemic factors, such as age and sex, it has limitations. The cross-sectional nature of the study limits our ability to assess longitudinal changes in mandibular cortical erosion and morphology. Future studies should explore these relationships longitudinally to better understand the progression of cortical bone degradation over time. Additionally, while panoramic radiography is a widely used and accessible tool, it lacks the precision of DXA in diagnosing osteoporosis. Thus, combining panoramic radiographic findings with DXA measurements in future studies could provide a more comprehensive approach to osteoporosis screening.

## 5 Conclusions

This study demonstrates that age, sex, and mandibular morphometric parameters significantly influence MCW and cortical erosion, with older individuals and women showing greater cortical degradation. These findings underscore the importance of panoramic radiography as a simple, cost-effective tool that could aid in the early detection of osteoporosis, particularly among high-risk populations such as older adults and postmenopausal women. By incorporating mandibular cortical measurements into routine dental examinations, dentists could play a pivotal role in identifying patients who may require further osteoporosis assessment and preventive care. This approach may improve patient outcomes by facilitating earlier diagnosis and intervention, thereby reducing the long-term burden of osteoporotic fractures. Mandibular morphometric measurements, such as RH, BW, and GA, are strongly associated with cortical changes, suggesting that panoramic radiography could be a valuable tool for early osteoporosis screening.

## Data Availability

The raw data supporting the conclusions of this article will be made available by the authors, without undue reservation.
